# Physicochemical and Rheological Properties of a Transparent Asphalt Binder Modified with Nano-TiO_2_

**DOI:** 10.3390/nano10112152

**Published:** 2020-10-28

**Authors:** Iran Rocha Segundo, Salmon Landi, Alexandros Margaritis, Georgios Pipintakos, Elisabete Freitas, Cedric Vuye, Johan Blom, Tom Tytgat, Siegfried Denys, Joaquim Carneiro

**Affiliations:** 1 Department of Civil Engineering, University of Minho, 4800-058 Guimarães, Portugal; efreitas@civil.uminho.pt; 2 Federal Institute Goiano, Rio Verde 75901-970, Brazil; salmon.landi@ifgoiano.edu.br; 3 Energy and Materials in Infrastructure and Buildings (EMIB) Research Group, University of Antwerp, 2020 Antwerp, Belgium; alexandros.margaritis@uantwerpen.be (A.M.); georgios.pipintakos@uantwerpen.be (G.P.); cedric.vuye@uantwerpen.be (C.V.); johan.blom@uantwerpen.be (J.B.); 4 Research Group Sustainable Energy, Air and Water Technology, University of Antwerp, 2020 Antwerp, Belgium; tom.tytgat@uantwerpen.be (T.T.); siegfried.denys@uantwerpen.be (S.D.); 5 Centre of Physics, University of Minho, 4800-058 Guimarães, Portugal; carneiro@fisica.uminho.pt

**Keywords:** asphalt binder, transparent binder, nanomaterials, TiO_2_, viscoelastic properties, FTIR, photocatalytic asphalt, light-colored asphalt, self-cleaning

## Abstract

Transparent binder is used to substitute conventional black asphalt binder and to provide light-colored pavements, whereas nano-TiO_2_ has the potential to promote photocatalytic and self-cleaning properties. Together, these materials provide multifunction effects and benefits when the pavement is submitted to high solar irradiation. This paper analyzes the physicochemical and rheological properties of a transparent binder modified with 0.5%, 3.0%, 6.0%, and 10.0% nano-TiO_2_ and compares it to the transparent base binder and conventional and polymer modified binders (PMB) without nano-TiO_2_. Their penetration, softening point, dynamic viscosity, master curve, black diagram, Linear Amplitude Sweep (LAS), Multiple Stress Creep Recovery (MSCR), and Fourier Transform Infrared Spectroscopy (FTIR) were obtained. The transparent binders (base and modified) seem to be workable considering their viscosity, and exhibited values between the conventional binder and PMB with respect to rutting resistance, penetration, and softening point. They showed similar behavior to the PMB, demonstrating signs of polymer modification. The addition of TiO_2_ seemed to reduce fatigue life, except for the 0.5% content. Nevertheless, its addition in high contents increased the rutting resistance. The TiO_2_ modification seems to have little effect on the chemical functional indices. The best percentage of TiO_2_ was 0.5%, with respect to fatigue, and 10.0% with respect to permanent deformation.

## 1. Introduction

For specific applications, it is important to control the light absorption and thermal energy storage in asphalt pavements, which can be carried out by the application of light-colored pavements [1]. Light and heat are essential influencing factors for asphalt pavements. Firstly, it is well known that they are essential keys for the asphalt binder aging, causing damage to asphalt roads [2,3]. The absence of light profoundly affects the visibility conditions, decreasing safety [1]. In contrast, a large amount of heating can increase the Urban Heat Island (UHI) effect in urban areas [4]. The conventional black color of asphalt pavements absorbs light and stores a large amount of thermal energy.

According to the World Health Organization (WHO), more than 90% of the global population lives in places where the concentrations of pollutants exceed their limits, presenting, as consequences, intensification of the greenhouse effect, acid rain, and public health problems, for example. The indispensable urgency for the reduction of air pollutants is clear from different scales and needs. As such, the introduction of semiconductor nanoparticles into asphalt mixtures can make part of the solutions available to mitigate air-quality problems [5,6,7,8,9,10,11].

It is estimated that 40% of urban areas are covered by pavements due to the rapid human development, affecting the local ecosystems and the subjacent surface conditions. Nowadays, another urban problem is the UHI phenomenon, which is the increase of temperatures in cities in comparison to the colder conditions of suburban zones and rural areas, due to the massive development of urbanization [4,12]. Traditional (asphalt) pavements and roofs absorb and store most of the solar energy during the day, which is released in the form of heat during the night. The dark surfaces of, for example, asphalt pavements are characterized by a sunlight reflection up to only 20%. Therefore, light-colored road pavements can be considered a viable technology to tackle this phenomenon. Additionally, these surfaces reduce the heat convection from pavement to air with a consequent decrease of ambient air temperature. Its high reflectivity reduces the overheating during the summer period, resulting in less distress and increased pavement durability [1,3].

A common practice for separating asphalt binder fractions is to fractionate it based on polarity using different solvents. By this method, the following fractions are obtained: asphaltenes, resins, aromatics, and saturates with decreasing polarity order [13]. To obtain light-colored asphalt pavements, transparent binders can be used. Thus, they are produced through three different processes: (i) bitumen modification based on the extraction of asphaltenes that are responsible for the black color of bitumen; (ii) synthetic binder production by transparent polymer materials; and (iii) blending specific resins with bio-oils or organic vegetal origin materials [1,3]. Even though they are not bituminous materials, their rheological properties are similar to asphalt bitumens [1]. They can even contribute to electricity cost savings (and reduced pollutant emissions) due to the increased visibility in dark areas, for example, in tunnels, and, consequently, a reduced need for lighting [1].

Hitherto, little research has been focused on the physicochemical and rheological properties of light-colored binders. Additionally, the use of TiO_2_ can bring two benefits in this sense besides environmental effects: (i) the functionalization by providing photocatalytic capability can contribute to the environmental remediation; and (ii) development of lighter asphalt mixture, e.g., from dark brown to light yellow, depending on the used granulates, which can mitigate the UHI. 

The literature presents a small number of studies addressing the use of the transparent binder. For example, Bocci et al. (2012) produced a light-colored asphalt mixture with conventional aggregates, lime filler, light-colored binder, and TiO_2_ powder (1% by aggregate weight). The coefficient of reflection related to night visibility and the luminance of this technology were much higher than those from the conventional asphalt mixture [1]. Bocci E. and Bocci M. (2014) continued their research on this subject, showing that light-colored dense-graded mixtures have similar mechanical properties when compared to the conventional asphalt mixture. They concluded that the light-colored asphalt pavement presented very high photometric properties even after five months from the traffic opening [14]. Sengoz et al. (2017) investigated the rheological properties of transparent binder in comparison to a traditional black bitumen. They concluded that the transparent and the traditional black bitumen had a similar performance [3].

On the basis of a review of the previous literature, there is a gap considering the rheological behavior of transparent binders modified with nano-TiO_2_. The use of these two materials together would combine their benefits into one single product: an asphalt pavement capable to photodegrade pollutants and avoid the UHI. The main objective of this research is to analyze the physicochemical and rheological properties of a transparent binder modified with nano-TiO_2_ (0.5%, 3.0%, 6.0%, and 10.0%) for the understanding of its limitations and definition of suitable destinations. For this, its physical (conventional), rheological, and chemical properties were assessed and compared to those of a conventional asphalt binder and a commercial PMB.

## 2. Materials

### 2.1. Binders

In this research, the transparent binder Kromatis 50/70 from Total (Rives-en-Seine, France) was used. According to the supplier, this light-colored synthetic binder presents properties similar to other bitumens. It is produced with hydrocarbon resins and low content of asphaltenes, which are removed and replaced with new elastomeric polymers [15].

A conventional 50/70 bitumen and a polymer-modified binder (PMB) (SBS-modified bitumen) were also used in this study. These reference binders were named as N50/70 and PMBTS, respectively.

### 2.2. TiO_2_ Nanoparticles

The semiconductor selected to provide multifunctional properties was the nano-TiO_2_ by Quimidroga (Aeroxide TiO_2_ P25) (Barcelona, Spain). Its main properties are 80% anatase and 20% rutile crystalline phases, purity > 99.5%, and particle size about 23 to 28 nm.

### 2.3. Sample Preparation

The nanoparticles were incorporated into the binder (at 150 °C for 30 min in a low shear mixer with a rotational speed of 1500 RPM) with four different contents: 0.5, 3.0, 6.0 and 10.0% (in the mass of the binder) with a similar modification procedure adopted in the literature review [10,16,17,18]. The particles were placed when the low shear mixer was working, homogenizing the binder already heated to 150 °C. All safety precautions were taken, considering personal protective equipment, engineering control (ventilated enclosures), and hygiene, among other things.

**Figure 1:**
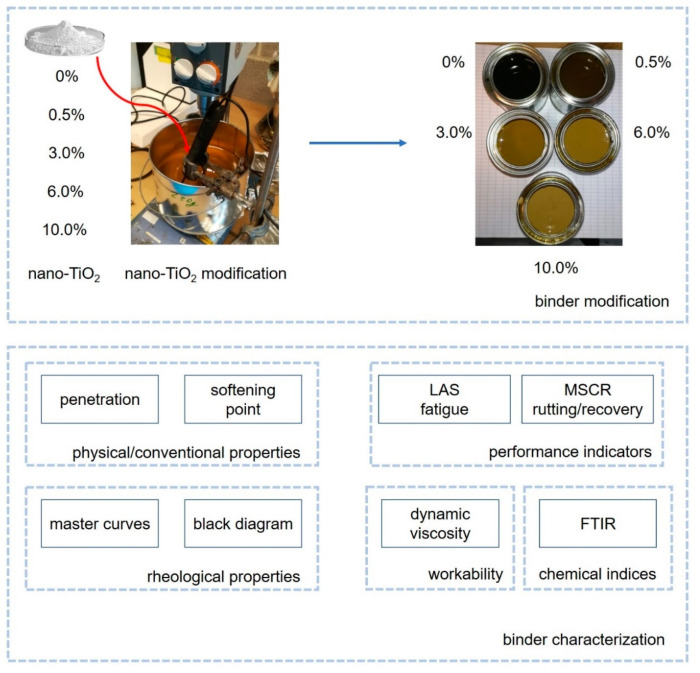
Schematic representation of this research.

The samples were named by the modification content: 0.5%, 3.0%, 6.0% and 10.0%. With the introduction of nano-TiO_2_, the color of the binder changes from dark brown (0%) to light yellow (10.0%), see Figure 1. One blend was prepared for each content. Regarding the performed tests, the number of replicates respected the requirements mentioned in the relevant European standard.

## 3. Methods

Different tests were carried out, such as penetration, softening point, Dynamic Shear Rheometer (DSR) tests (complex modulus, Linear Amplitude Sweep—LAS, Multiple Stress Creep Recovery—MSCR), Dynamic viscosity and Fourier Transform Infrared (FTIR) spectroscopy, in order to determine conventional, rheological and chemical properties. Figure 1 presents the schematic summary of the preparation and characterization methodology adopted in this paper. 

### 3.1. Penetration and Softening Point

Penetration and softening point were tested according to EN 1426/2015 and EN 1427/2015, respectively. They indicate the basic properties of asphalt binders.

### 3.2. Dynamic Viscosity

A dynamic viscosity test was carried out following the EN 13302/2010 standard, but only for the transparent binders (with and without nano-TiO_2_). The objective was to evaluate the workability of the binders according to Superpave specifications. The highest allowed viscosity to respect the workability is 3 × 10^3^ cP (or 3 Pa·s) at 135 °C [19,20].

### 3.3. Viscoelastic Behavior

The viscoelastic behavior of the binders was assessed using the Dynamic Shear Rheometer (DSR). The DSR used in this study is an Anton Paar MCR 500 (Graz, Austria). For temperature ranges from 0 °C to +40 °C and from +40 °C to +80 °C, the 8 mm and 25 mm plate geometries were used, accordingly, as described in EN 14770:2012. For each temperature step (increments of 10 °C), frequency sweep tests were performed (0.1–10 Hz) on two replicates per binder sample, within the linear viscoelastic region (LVER) of the binders. The data were further analyzed using the RHEA™ software (v2.0, Abatech, Blooming Glen, PA, USA) [21]. The shifting of the data was performed using the Gordon and Shaw procedure [22]. The phase angle and complex modulus master curves are presented in their original format, without fitting any mathematical or mechanical models. 

Black diagrams (complex modulus versus phase angle) aim to identify discrepancies of the rheological data, breakdown of time-temperature equivalence, and thermo-rheological simplicity [23]. A smooth curve indicates time–temperature equivalence, a typical response of unmodified binders. On the other hand, discontinuities indicate the presence of high wax content bitumen, highly polymer modified bitumen, or a highly asphaltene structured binder [23]. Additionally, it is possible to notice whether there are different dominances when the binder is a composite [23]. This phenomenon happens, for example, when the complex modulus trend changes with the increase of the phase angle, a phenomenon known as curling.

### 3.4. Fatigue Resistance (LAS Test)

To evaluate the fatigue resistance of bituminous binders, the Linear Amplitude Sweep (LAS) test was performed. This test is an accelerated method that uses the DSR (8 mm parallel plate geometry at 15 °C), which consists of two steps: (i) firstly, a frequency sweep test, and (ii) secondly, an amplitude sweep test, as described in AASHTO TP 101-14. The frequency sweep test (0.2–30 Hz) is used to define the undamaged properties and fatigue law parameters, at a strain level of 0.1%. A linear amplitude sweep test is performed at 10 Hz, and the strain amplitude is linearly increased over 3000 cycles, from 1% to 30%. The Viscoelastic Continuum Damage (VECD) theory is used to determine the parameters *A* and *B* of the fatigue law (Equation (1)) [24], in order to determine the fatigue life (*Nf*). The failure point is determined as the point when the product of the complex modulus (G*) and phase angle (*δ*) sine is reduced by 35% from its initial value.

(1) $$Nf=A\;\gamma^{B}$$

Both the fatigue curves and the Nf for strain levels (γ) equal to 2.5% and 5%, related to a strong and weak pavement [25], respectively, will be presented.

### 3.5. Rutting Resistance Indicator (MSCR Test)

The Multiple Stress Creep Recovery (MSCR) was performed using the DSR together with the 25 mm plate and 1 mm gap, as described in EN 16659:2015. The test was performed at 50 °C, at two different stress levels (0.1 and 3.2 kPa) over ten load cycles. Each cycle consists of 1 s loading followed by 9 s of a recovery period, from which two parameters are obtained: (i) the non-recoverable creep compliance *Jnr* (Pa^−1^), which is the ratio between the residual strain and the stress applied; and (ii) the recovery *R* (%), showing proportionally how much strain the sample recovers at the end of the cycle. *R* (%) can be used to identify the presence of polymer modifications in the asphalt binders. 

### 3.6. FTIR

Chemical characterization of binders has received attention in the literature as it can present functional groups related to the crude oil origin, the polymer modification, and the degree of oxidation [26,27,28,29,30,31]. Since this paper aims to analyze the chemical characteristics of the transparent binder modified with nano-TiO_2_, three approaches were carried out: (i) identification of FTIR peaks; (ii) establishment of a possible relationship between the TiO_2_ modification level and related chemical groups; and (iii) comparison of standard indices with reference binders used in this study.

The Thermo Scientific Nicolet iS10 Fourier Transform Infrared (FTIR) spectrometer (Waltham, MA, USA) is equipped with an Attenuated Total Reflectance (ATR) fixture and a Smart Orbit Sampling Accessory. The average spectra were obtained after the acquisition of the spectra, 32 repetitive scans in the range 400 cm^−1^ to 4000 cm^−1^ with a resolution of 4 cm^−1^ were performed to deliver an average spectrum. A hot droplet of each binder was placed on the crystal, and its respective spectrum was measured. 

The chemical structure of the binders was analyzed using indices, *I*, from the obtained FTIR spectrum [32]. Each *I* (Equation (2)) is calculated by the ratio of the peak area of the identified band by the total area (Equation (3)) of the spectrum.

(2) $$I_{Functional\;Group}=\frac{A_{i}}{\Sigma A}$$

(3) $$\begin{array}{c} \Sigma A=A_{1700}+A_{1600}+A_{1460}+A_{1376}+A_{1030}+A_{864}+A_{818}+A_{743}+A_{724}\\ +A_{\left(2953,2923,2862\right)} \end{array}$$

where $A_{i}$ is the peak area of the specific functional group.

The areas defined by the introduced baselines and the part of the spectrum were calculated using a specific software Origin. Each peak is attributed to a functional group remaining unaffected during service life, but also to groups responsible for aging or polymer presence [26,28]. When it comes to the groups responsible for TiO_2_, echoing [33,34,35] in the region below 1000 cm^−1^, several peaks are ascribed to TiO_2_ presence. Previous researchers have demonstrated that the peak around 657 cm^−1^ is attributed to Ti-O-Ti stretching vibration, whereas the peak around 590 cm^−1^ is due to the vibration of Ti-O-O. A broader band of wavenumbers was calculated around these peaks in order to capture their increase by elevating the TiO_2_ modification level. It should be noted that a horizontal baseline coinciding with the *X*-axis was used for the calculation of this area. *RI* (Equation (4)) was calculated in order to check the relative increase of TiO_2_ modification. $I_{\mathrm{TI}-O+\mathrm{Ti}-O-O}$ is the index of each binder, and I_TI-O+Ti-O-O0%_ is the index of the transparent base binder (0%).

(4) $$RI=\frac{I_{\mathrm{TI}-O+\mathrm{Ti}-O-O}-I_{\mathrm{TI}-O+\mathrm{Ti}-O-O0\%}}{I_{\mathrm{TI}-O+\mathrm{Ti}-O-O0\%}}\;\%$$

More specifically, for asphalt binders, the sulfoxide, carbonyl, aromatic, aliphatic, branched aliphatic, long chains, polybutadiene, and polystyrene indices were calculated. Sulfoxide and carbonyl indices are both related to aging. 

They were calculated considering the following method: (i) aromatic index: A_1600_/ΣA; (ii) aliphatic index: (A_1460_ + A_1376_)/ΣA; (iii) branched index: A_1360_/(A_1460_ + A_1376_); (iv) long chain index: A_724_/(A_1460_ + A_1376_); (v) carbonyl index: A_1700_/ΣA; (vi) sulphoxide index: A_1030_/ΣA; (vii) polybutadiene index: A_966_/ΣA; and (viii) polystyrene index: A_699_/ΣA.

Aromatic, aliphatic, branched aliphatic, and long chains are the structural groups of asphalt binders. Polybutadiene and polystyrene are associated with SBS modified binders. For details concerning the determination of standard indices related to oxidative aging of the binder, the reader is referred to the protocol described in [32]. Briefly, a common practice in this processing method is to introduce tangential baselines defined by limits around certain peaks [36].

## 4. Results and Discussion

### 4.1. Penetration and Softening Point

**Figure 2:**
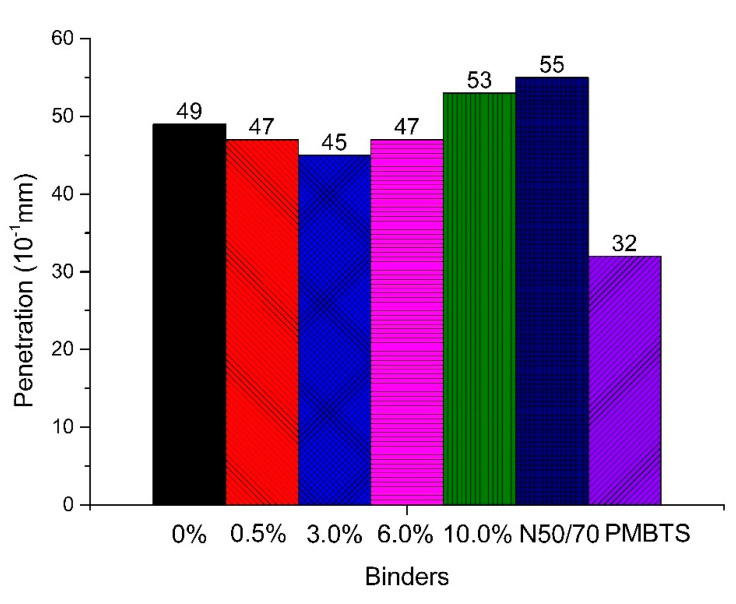
Penetration results of the binders of this study.

**Figure 3:**
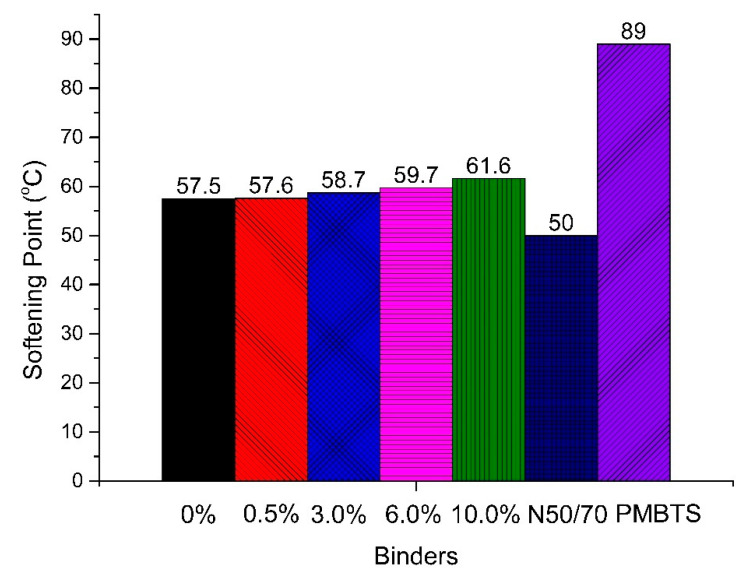
Softening point results of the binders of this study.

Figure 2 and Figure 3 show the resulting penetration and softening point of the studied binders. When compared to the transparent base binder, the inclusion of 0.5%, 3.0% and 6.0% TiO_2_ nano-modification decreased the penetration from 49 to 47, 45, and 47 × 10^−1^ mm, respectively. For 10.0% TiO_2_, it increased to 53 × 10^−1^ mm, which is similar to the N50/70 results. When compared to the PMBTS, all the penetration results were higher. Therefore, the penetration values of the Kromatis binder were between the conventional and the PMB binders.

For the softening point, the results of the transparent binders were around 59 °C. The increase of the TiO_2_ content gradually increased the softening point by about 4 °C. The transparent binders had a softening point again between those of the conventional and the PMB binders.

Comparing these results to those obtained by Sengoz et al. (2017) for the same binder from the same supplier, they showed that the penetration and softening points were 55 × 10^−1^ mm and 56 °C [3]. Thus, in this research, the transparent base binder had a lower penetration but a higher softening point [3].

It can be concluded that the transparent binders had results between those of the conventional and the PMB binders, but closer to those of the conventional one. The incorporation of nano-TiO_2_ gradually increased the softening point. For the penetration, the results were lower until 6.0%, but higher for 10.0%.

### 4.2. Dynamic Viscosity

**Figure 4:**
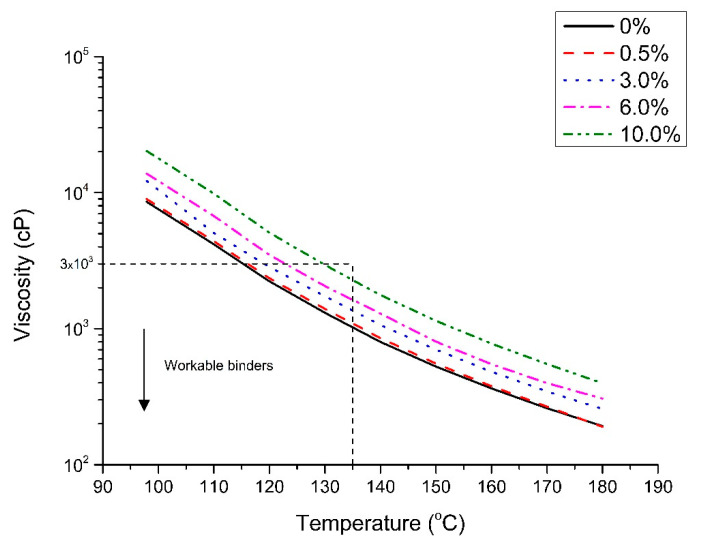
Dynamic viscosity results of the binders of this study.

The dynamic viscosity, determined only for the transparent binders, is shown in Figure 4. The introduction of nano-TiO_2_ increased dynamic viscosity. At 135 °C, the viscosity increased from 1 × 10^3^ cP (for the base binder) to 2.3 × 10^3^ cP (for the 10.0%). Additionally, all the binders had a viscosity lower than 3 × 10^3^ cP, considered as the recommended maximum viscosity criterion under Superpave to guarantee proper binder pumping in the asphalt plant during production [37]. If this value is higher than 3 × 10^3^ cP, excessive energy is needed for the mixing and compaction of asphalt mixtures [19]. It can be concluded that all the modified transparent binders using the contents of nano-TiO_2_ studied (from 0 to 10.0%) are feasible with respect to their workability. It is also interesting that the 10.0% TiO_2_ appears to have the highest energy requirements as its viscosity is close to the Superpave threshold. Thus, from an economic point of view, it would be favorable to target lower TiO_2_ levels.

In addition, the comparison of the results to those from the literature reveals that a higher viscosity was reached (1 × 10^3^ cP). For example, Sengoz et al. (2017) presented 788 cP in dynamic viscosity for the same transparent binder [3].

### 4.3. Viscoelastic Behavior

**Figure 5:**
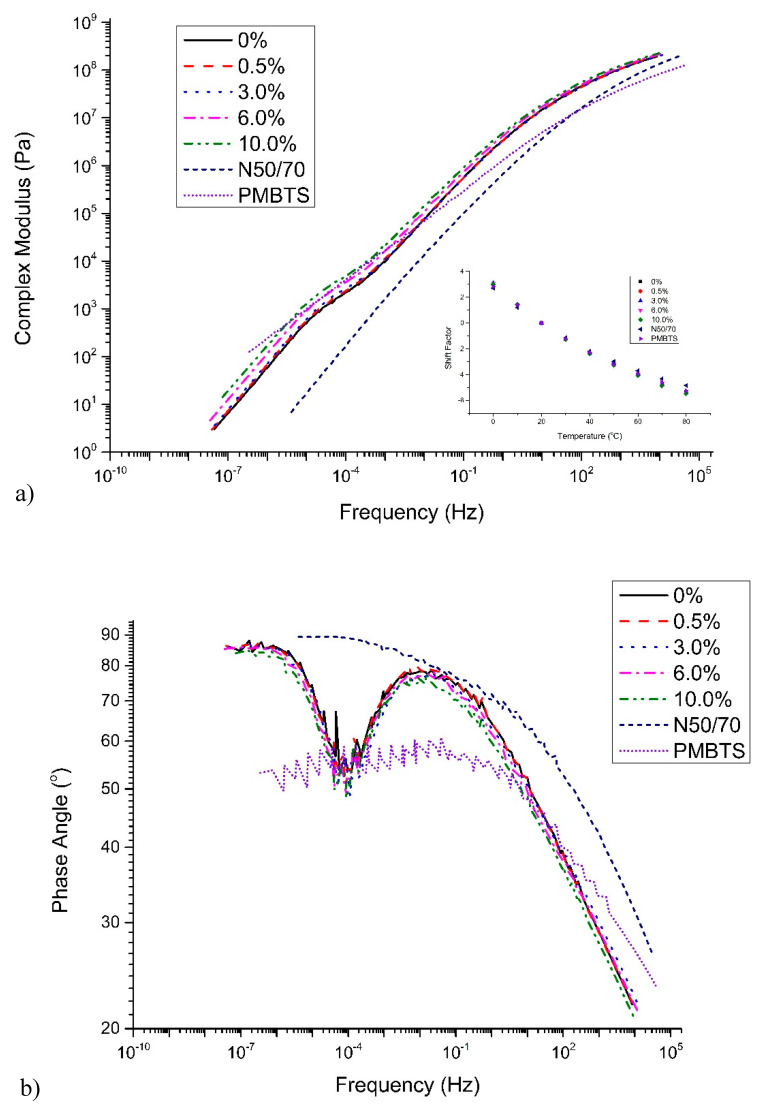
(**a**) Complex modulus and (**b**) phase angle master curves of the binders of this study.

The complex modulus and phase angle master curves are presented in Figure 5a,b. The addition of TiO_2_ slightly alters the viscoelastic behavior of the transparent binder, leading to a simultaneous small increase of modulus and a decrease of the phase angle. The N50/70 shows a simple viscoelastic behavior with the phase angle gradually approaching the viscous asymptote of 90° at elevated temperatures, a typical response of an unmodified binder. Concerning the complex modulus, the N50/70 shows similar values to the PMBTS at frequencies above 10 Hz. At low frequencies (related to high temperature), N50/70 shows the lowest complex modulus compared to other binders, which was an expected observation, since the modulus of those binders is greatly influenced by the polymer modification.

Comparing the transparent binder with the PMBTS, it can be seen that the complex modulus is similar at lower frequencies, while the PMBTS demonstrates a lower modulus at frequencies above 0.01 Hz. Looking at the phase angle, both reveal the presence of elastomeric modification, which is visible by the drop of the phase angle at a low reduced frequency. For the PMBTS, the dominance of the polymeric phase starts below 1 Hz and shows at a steady plateau stage of 60°. On the other hand, the dominance of the polymeric phase for the transparent binders starts after 0.01 Hz (which can be translated that the polymer network is “active” at higher temperatures compared to the PMBTS), showing a significant drop of the phase angle and then gradually approaching the viscous asymptote of 90°.

The last part shows that the polymer network is no longer dominant. Those distinct differences can be attributed to the difference between the base binders as well as the compatibility between base and polymer [38]. Another possibility is the thermal history of the binders, which can significantly influence the rheological behavior of elastomeric binders, as demonstrated by Soenen et al. [39].

**Figure 6:**
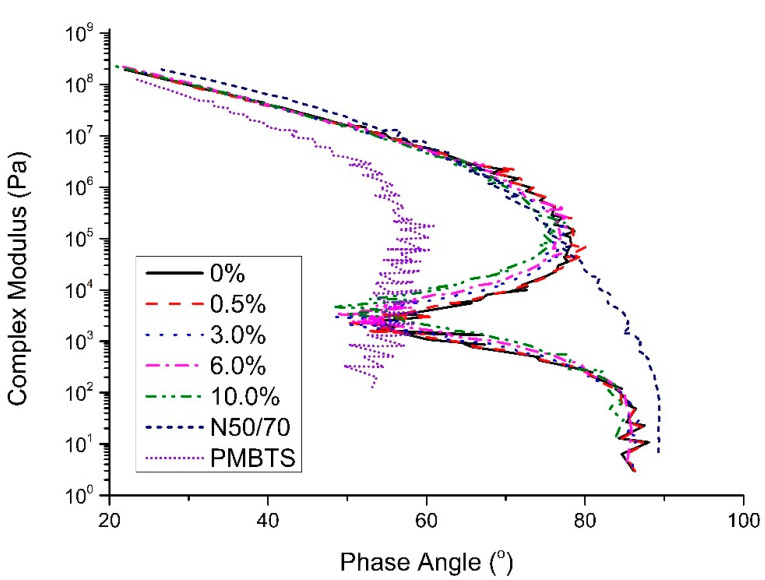
Black diagram of the evaluated reference and transparent binders.

The black diagram (Figure 6) shows the combined effect of complex modulus and phase angle for the different binders. The N50/70 binder presents a conventional black diagram curve. Therefore, it is smooth, and the complex modulus decreases while the phase angle increases. The presence of a polymer can be seen by the shift of the curve towards a lower phase angle (higher elastic behavior) [23]. More specifically, for the PMBTS, the plateau near 60° (at a temperature of 58 °C) can indicate that the polymer forms a continuous elastic network when dissolved in bitumen, making it a polymer-dominant phase.

The shape of the curve of the transparent binders is different from the reference ones, showing three distinctive regions: (i) from 10^8^ to 5 × 10^5^ Pa, the complex modulus decreases with the increase of the phase angle; (ii) from 5 × 10^5^ to 5 × 10^4^ Pa, the complex modulus decreases when the phase angle increases; and (iii) from 5 × 10^4^ Pa, with the same pattern to that of the first region, including the shape. This viscoelastic response indicates an alternation of dominance between the materials that compose the transparent binders, as also observed in the phase angle master curve. Additionally, transparent binders seem to be more elastic than N50/70, with a partial shift towards the left. The addition of TiO_2_ seems to have a small, rather insignificant, effect on the viscoelasticity of the transparent binder. Based on the observations of the master curves and black diagram, the addition of TiO_2_ up to 10.0% seems not to introduce any noticeable effect on the structure of the transparent binders. 

### 4.4. Fatigue Resistance (LAS Test)

**Figure 7:**
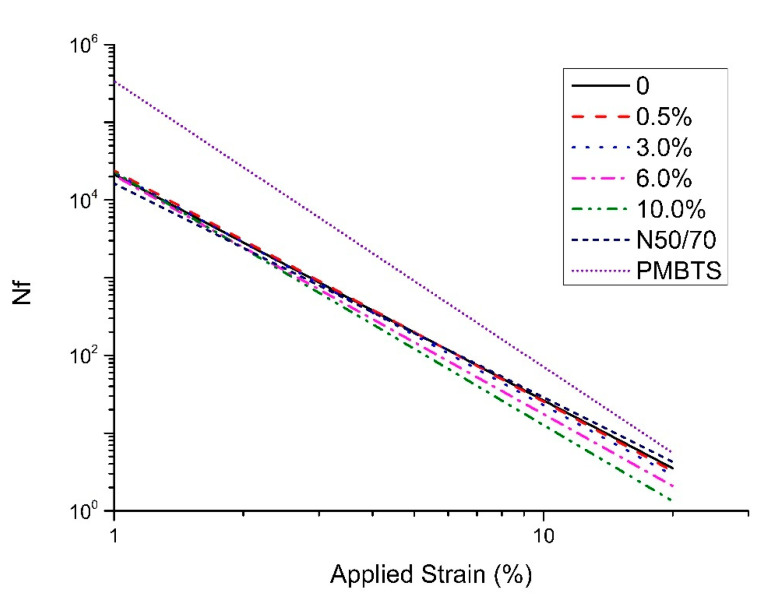
LAS Test: Nf versus applied strain.

The fatigue curves of each binder are presented in Figure 7 and the corresponding parameters are further elaborated in Table 1.

The PMBTS shows the highest fatigue resistance among the binder samples, while the transparent base binder and the N50/70 showed similar results. The addition of TiO_2_ seems to harm the fatigue life of the transparent binder, except for the 0.5% dosage, where it shows a slightly improved fatigue life. In more detail, the addition of TiO_2_ leads to a proportional decrease of the slope (parameter B), while no clear trend is evident on the effect of TiO_2_ on the intercept (parameter A).

For all the binders, from the lowest to the highest fatigue performance considering the applied strain of 2.5% (representative strain level of a “weak” pavement structure), the progressive sequence is 10.0% < 6.0% < N50/70 < 3.0% < 0% < 0.5% < PMBTS. For the applied strain of 5% (representative strain level of a “strong” pavement structure), the progressive sequence is 10.0% < 6.0% < 3.0% < N50/70 < 0% < 0.5% < PMBTS. High contents of nano-TiO_2_ slightly reduced the binder fatigue resistance. Additionally, higher differences are found for an applied strain level of 5%, related to the strong pavement structures.

### 4.5. Rutting Resistance Indicator (MSCR Test)

The MSCR test was introduced as a test to evaluate the resistance to permanent deformation (rutting) as well as a tool to evaluate the quality of polymer modified binders [40,41]. Generally, a combination of high recovery (*R*) and low non-recoverable compliance (*Jnr*) indicates a good quality PMB that can be used in high traffic pavements, as described in AASHTO M332. Such limits that distinguish between the acceptance levels for different traffic levels have not been established by the EN 16659. Therefore, a comparative evaluation of the rutting resistance of the binders in this study is presented. 

The MSCR test results are presented in Table 2. Considering the %*R*, the transparent binders exhibit a recovery between the reference binders (higher than N50/70 but lower than PMBTS), but with values closer to PMBTS. At a stress level of 100 Pa, while the conventional binder N50/70 demonstrates recovery of only 9.0%, the transparent binders show at least 63.6% and the PMBTS 82.1%.

The incorporation of nano-TiO_2_ increased the *R100* for the contents 6.0% and 10.0% when compared to 0%. The contents 0.5% and 3.0% had similar *R100* to 0%. For *R3200*, the transparent binders had similar results, from 63.2% to 65.8%, for 0% and 10.0% TiO_2_ addition respectively.

Regarding the *Jnr* values, again, the transparent binders show an intermediate behavior between the reference binders N50/70 and PMBTS. The incorporation of nano-TiO_2_ decreased the *Jnr* (100 and 3200 Pa^−1^) for the contents 3.0%, 6.0% and 10.0% when compared to 0%. As can be expected, the content 0.5% had a similar *Jnr* to 0% (transparent base binder).

Sengoz et al. (2017) analyzed the same Kromatis 50/70 from the same supplier. They indicated values of R% between the N50/70 and PMBTS as well, 27.6% and 16.6% for 100 Pa and 3200 Pa, respectively [3]. Nevertheless, their results are closer to the N50/70 than the PMBTS. Considering *Jnr*, the results from Sengoz et al. (2017) are much higher than those obtained in this research for all the binders (base, modified, and reference ones).

It can be concluded that the transparent binders present better rutting resistance than the conventional N50/70 with higher recovery and lower non-recoverable creep compliance. This fact was expected, since the transparent binder contains elastomeric polymers, as demonstrated earlier in Section 4.3. However, the transparent binders presented lower rutting resistance than the PMBTS. The incorporation of nano-TiO_2_ can increase the rutting resistance for high contents (mainly 6.0% and 10.0%).

### 4.6. FTIR

#### 4.6.1. Peak Identification

**Figure 8:**
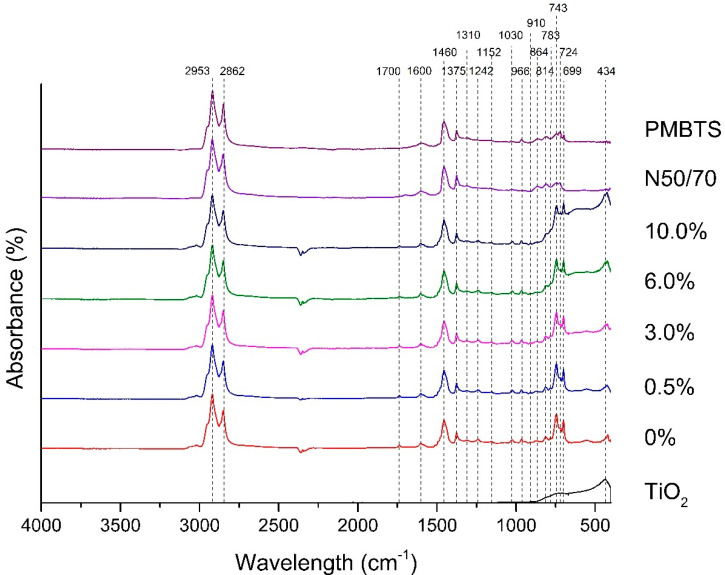
FTIR spectra of the binders of this study.

The FTIR spectra (absorbance versus wavelength) of the (TiO_2_-modified) transparent binders, the reference binders, and the pure TiO_2_ are shown in Figure 8. Peaks at 2953 cm^−1^ and 2862 cm^−1^ are associated with stretching vibrations of *sp^3^* C-H in aliphatic chains, as asymmetric and symmetric stretches, respectively. Peaks at 1460 cm^−1^ are characteristic of bending vibrations of methylene groups (-(CH_2_)_n_). The peak at 1375 cm^−1^ is attributed to the bending of methyl groups (-CH_3_), which is related to aliphatic branched bands. The long-chain band can be seen at 724 cm^−1^, associated with the rocking motion of -CH_2_ groups in an aliphatic chain. The peak at 1700 cm^−1^ is related to the stretching of carbonyl band C=O typical of carboxylic acids [42], being one of the most important peaks for the asphalt binder aging [43,44,45].

Stretching absorptions of C=C bond in aromatic rings occur at the peaks at 1600 cm^−1^. The peaks that appear between 910 cm^−1^ and 699 cm^−1^ can be analyzed carefully to show ortho-, meta- and para-disubstituted rings presented in aromatic compounds and are associated with out-of-plane C-H bending vibrations in this structure. Due to the complex composition of the asphalt binders, all these compounds may be presented in the analyzed samples. For example, the pair 743 cm^−1^ and 699 cm^−1^ can match with monosubstituted rings; a single peak at 743 cm^−1^ may be associated with 1,2-disubstituted rings; peaks at 864 cm^−1^, 783 cm^−1,^ and 699 cm^−1^ may be attributed to 1,3-disubstituted rings; and finally, the pair 864 cm^−1^ and 814 cm^−1^ can match to 1,4-disubstituted rings [43,44,45].

The peaks at 966 cm^−1^ and 699 cm^−1^ may still correspond to the bending out-of-plane of C-H of trans-alkenes (from polybutadiene) and C-H out-of-plane bending in monoalkylated aromatics (from polystyrene) associated with SBS. The peak at 910 cm^−1^ can also show terminal-alkenes [43]. The peak at 1375 cm^−1^ may be associated with the asymmetric stretch of sulfonyl chlorides S=O bond. While the peaks at 1310 cm^−1^ and 1152 cm^−1^ are typical of respectively asymmetric and symmetric stretches of sulfones S=O bonds [13]. At 1030 cm^−1^, there is the other peak directly related to asphalt binder aging, the stretch of sulfoxide S=O bond [43,44,45]. The peak 1242 cm^−1^ can be linked to the asymmetric stretching vibration of sulfate esters [13].

For the transparent binders, the peak 434 cm^−1^ represents a stretching vibration of metal oxides (M-O) bond (M can be Si, Mn, V, Ni, and others) [46,47,48,49]. This peak (434 cm^−1^) is also attributed to stretch absorptions of Ti-O bond [50], and the increase in this peak area in the TiO_2_-modified transparent binders may indicate the proper incorporation of the semiconductor in the asphalt binder.

#### 4.6.2. Relationship between the TiO_2_ Modification Level and the Chemical Responsible

**Figure 9:**
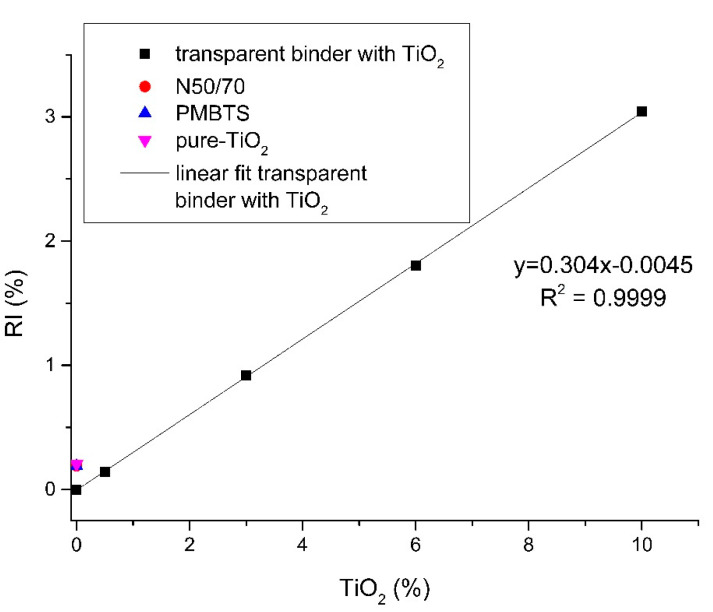
Correlation of the nano-TiO_2_ modification level with the increase of the TiO_2_-related index.

Figure 9 shows the graph *RI* versus % TiO_2_. It can also be seen that the addition of nano-TiO_2_ increases the relative increase *RI* (from TiO_2_ related vibration area). A linear correlation of the nano-TiO_2_ percentage and the *RI* can be found. To some extent, this shift of the spectra with the addition of nano-TiO_2_ is to be expected, as the vibrations become more evident when the binder is more diluted. The high correlation coefficient (R^2^ = 0.99) of the increase of this index with the modification level (Figure 9) confirms the assumption that nano-TiO_2_ can be used as a marker in bituminous blends [51,52]. This analysis can be used in order to assess the presence of TiO_2_ and quantify its incorporation as a binder modifier.

#### 4.6.3. Relationship between the TiO_2_ Modification Level and the Chemical Responsible

**Figure 10:**
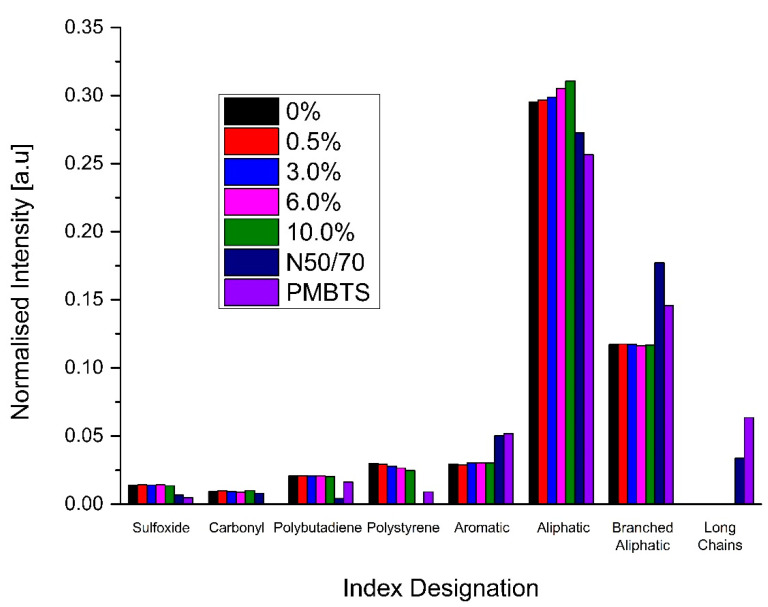
FTIR indices of the nano-TiO_2_-modified, base and reference binders.

The results of FTIR are presented in Figure 10 in terms of oxygen- and polymer-related indices. The chemical indices sulfoxide, carbonyl, polybutadiene, polystyrene, aromatic, aliphatic, branched aliphatic, and long chains are presented in terms of their normalized intensity. This study confirms that the initial sulfoxide and carbonyl levels of nano-TiO_2_-modified are similar to the reference unmodified binder N50/70 and the SBS-modified PMBTS. This observation gives rise to the assumption that the presence of nano-TiO_2_ does not introduce new functionalities in sulfoxide- or carbonyl-related groups such as esters, carboxylic acids, and ketones. Furthermore, the modification of the binder with nano-TiO_2_, according to other studies [53,54], can reduce the long-term oxidation performance due to its capability to reflect and absorb UltraViolet (UV) light during photocatalysis. It is also initially chemically neutral for the polar carbonyl groups.

A detailed observation of the two aging-related indices implies that, although of similar magnitude, slightly higher indices can be found compared to the two reference binders N50/70 and PMBTS. This fact can be explained to be the result of the different initial sulfur content for the sulfoxide index. In other words, the origin of the crude oils of the binders is different, and this has a clear implication for the S=O-containing groups. When examining the slightly higher carbonyl index of the nano-TiO_2_-modified binders, one can highlight two important points. Firstly, the binder used for modification with nano-TiO_2_ was different from the unmodified reference binder, and transparent binders show a carbonyl index of the same magnitude. The level of modification seems to have a negligible effect on the initial carbonyl index. That is, the increased carbonyl level can be attributed to the binder used as the base for modification and not the nano-TiO_2_ addition itself. Following the explanation provided for the higher carbonyl increase of nano-TiO_2_-modified binders, the same line of thought can be followed for the slightly lower aromatic, branched aliphatic, and long chains indices and the higher aliphatic index compared to the reference binders.

In parallel, as the introduction of elastomeric polymers produces the binder selected as the base for TiO_2_ modification, the polymer-related indices were evaluated. A comparison of the polybutadiene and polystyrene indices reveals that the binder used for modification (transparent base binder) is highly modified compared to the SBS modified reference binder PMBTS. For the binder N50/70, these indices are not applicable.

In contrast to the rheological parameters, the transparent binders did not show intermediate behavior between the reference binders N50/70 and PMBTS. They showed higher indices for sulfoxides, carbonyl, polybutadiene, polystyrene, and aliphatic but lower for aromatic, branched aliphatic and long chains. Their long chains were null. Moreover, the TiO_2_ modification seems to have little effect on the indices, except for the polystyrene and aliphatic indices. The polystyrene index decreases, and the aliphatic index increases with the increase of nano-TiO_2_.

## 5. Conclusions

A transparent binder modified with nano-TiO_2_ was characterized with respect to its physicochemical and rheological properties in this paper. This is useful for specific applications, such as tunnels, calming traffic areas, among others, as the nano-TiO_2_ provides to the pavement a photocatalytic function and better visibility. Modified transparent binders, with 0.5%, 3.0%, 6.0%, and 10.0% of nano-TiO_2_, were compared to the transparent base binder and two commercial asphalt binders (conventional and PMB).

Based on the results, the transparent binders (base and modified) performed similarly to the conventional binder and PMB, being workable in terms of their viscosity. The transparent binders clearly revealed the presence of an elastomeric modification, indicating polymer modification, and suggested an alternation of elastic/viscous behavior between the materials that compose the transparent binders.

The incorporation of nano-TiO_2_ gradually increased the softening point and decreased the penetration by up to 6.0% of modification, causing no substantial changes in the Complex Modulus of the transparent binder. There is no evidence that the addition of TiO_2_ up to 10.0% significantly affects the structure or visco-elastic behavior of the chemical indices, except for the polystyrene and aliphatic indices, of either of the transparent binders. Additionally, the addition of TiO_2_ would reduce the fatigue life of asphalt pavements using the transparent binder. On the contrary, it would increase the rutting resistance for high contents.

Transparent binders with TiO_2_ give promising results, based on their conventional, rheological, and chemical performance. On the one hand, the best percentage for the addition of TiO_2_, based on the results, without compromising the performance of the transparent binder, was 0.5% with respect to fatigue resistance. On the other hand, 10.0% nano-TiO_2_ was the best with respect to permanent deformation.

Although the objectives of this research were achieved, it is essential to carry out this analysis on more samples, as limited numbers were used. The next steps of this research are to evaluate the light-colored and photocatalytic pavements considering the properties of color and photocatalysis and analyze the aging performance of the transparent binders with nano-TiO_2_. Another topic that must be assessed is the analysis of the total life cycle cost (including environmental impact/benefits).

## Figures and Tables

**Table 1:** LAS test results.

Binder	Parameter			
	A	B	*Nf* 2.5%	*Nf* 5%
0%	21,348	−2.9	1486	198
0.5%	23,946	−3.0	1568	199
3.0%	22,578	−3.0	1453	182
6.0%	20,724	−3.1	1241	148
10.0%	22,942	−3.3	1160	121
N50/70	16,303	−2.8	1308	194
PMBTS	338,013	−3.7	11,631	909

**Table 2:** MSCR test results.

Binder	Parameter			
	*Jnr*, 100 (kPa^−1^)	*Jnr*, 3200 (kPa^−1^)	*R100* (%)	*R3200* (%)
0%	0.2	0.2	64.8	63.2
0.5%	0.2	0.2	63.6	62.3
3.0%	0.2	0.2	65.5	63.8
6.0%	0.1	0.1	69.9	64.4
10.0%	0.1	0.1	73.1	65.8
N50/70	0.6	0.6	9.0	6.0
PMBTS	0.0	0.0	82.1	85.7
Kromatis 50/70 from [3]	1.8	2.3	27.6	16.6
